# Development of a Propidium Monoazide-Based Viability Quantitative PCR Assay for Red Sea Bream Iridovirus Detection

**DOI:** 10.3390/ijms24043426

**Published:** 2023-02-08

**Authors:** Kyung-Ho Kim, Gyoungsik Kang, Won-Sik Woo, Min-Young Sohn, Ha-Jeong Son, Chan-Il Park

**Affiliations:** Department of Marine Biology and Aquaculture, College of Marine Science, Gyeongsang National University, 2, Tongyeonghaean-ro, Tongyeong 53064, Republic of Korea

**Keywords:** red sea bream iridovirus, propidium monoazide, viability qPCR, seawater concentration

## Abstract

Red sea bream iridovirus (RSIV) is an important aquatic virus that causes high mortality in marine fish. RSIV infection mainly spreads through horizontal transmission via seawater, and its early detection could help prevent disease outbreaks. Although quantitative PCR (qPCR) is a sensitive and rapid method for detecting RSIV, it cannot differentiate between infectious and inactive viruses. Here, we aimed to develop a viability qPCR assay based on propidium monoazide (PMAxx), which is a photoactive dye that penetrates damaged viral particles and binds to viral DNA to prevent qPCR amplification, to distinguish between infectious and inactive viruses effectively. Our results demonstrated that PMAxx at 75 μM effectively inhibited the amplification of heat-inactivated RSIV in viability qPCR, allowing the discrimination of inactive and infectious RSIV. Furthermore, the PMAxx-based viability qPCR assay selectively detected the infectious RSIV in seawater more efficiently than the conventional qPCR and cell culture methods. The reported viability qPCR method will help prevent the overestimation of red sea bream iridoviral disease caused by RSIV. Furthermore, this non-invasive method will aid in establishing a disease prediction system and in epidemiological analysis using seawater.

## 1. Introduction

Red sea bream iridovirus (RSIV), which is an enveloped deoxyribonucleic acid (DNA) virus belonging to the genus *Megalocytivirus* of the family *Iridoviridae* [1], infects several cultured and wild marine fish species and has caused significant economic damage to the fishing industry. It was first described in Japanese red sea bream (*Pagrus major*) in 1990 [2]. Since then, it has been detected in more than 30 cultured marine fish species [3]. RSIV causes red sea bream iridoviral disease (RSIVD) mainly reported in Southeast Asian and East Asian countries. Given its impact on fishery industries, RSIVD has been declared a reportable disease by the World Organization for Animal Health [3]. RSIVD mainly occurs during summer, when temperatures can reach 25 °C, and causes a high mortality of 60–100% in rock bream (*Oplegnathus fasciatus*), a highly susceptible fish species [3,4,5]. Recently, a correlation has been found between the progress of RSIV infection in rock bream and the viral load shed into seawater [6]. The horizontal transmission of infection through water is considered the main route of the spread of RSIV infection in an aquatic environment [3,7,8]—the virus particles shed by infected fish can travel through seawater and infect nearby farmed fish [9]. Because there is no cure, early and rapid detection of RSIV is of great significance for disease prevention and control.

Several methods, such as cell culture [10], loop-mediated isothermal amplification [11], polymerase chain reaction (PCR) [12], and nested PCR [13], have previously been reported for RSIV detection. However, these assays cannot be used to quantitatively analyze RSIV and obtain information about disease progression. Recently, a quantitative PCR (qPCR) assay with high sensitivity and specificity has been developed for the quantitative detection of RSIV [14]. However, this assay does not effectively differentiate between infectious and inactive viruses. The DNA from inactive viruses can serve as a template in qPCR amplification, leading to the overestimation of infectious viruses in diseased fish. Additionally, RSIV-infected fish may continue to shed viral DNA [6]. However, persistent, positive qPCR results may not necessarily indicate infectivity. Therefore, an assay that can rapidly and conveniently differentiate between infectious and inactive viruses must be developed to control the horizontal transmission of RSIV.

The standard method for assessing the infectivity of RSIV is to use susceptible grunt fin (GF) cells to identify the typical cytopathic effect (CPE), which is characterized by the presence of round and enlarged cells [10,15]. However, complete CPE in RSIV-inoculated cells does not occur even in GF cells. Furthermore, a large amount of virus is required as an inoculum to observe CPE [15]. To overcome these shortcomings, a new molecular technique, called viability qPCR assay, has recently been developed wherein survival markers such as propidium monoazide (PMAxx) dye and metal compounds are incorporated into qPCR-based methods [16,17,18,19,20]. This novel assay involves the uses of a membrane-impermeable material that can only enter damaged or destroyed capsids. Furthermore, a molecular approach based on viability qPCR has been applied to assess capsid integrity [21,22,23]. However, the utility of this technique for differentiating between infectious and inactive RSIV has not been examined.

In this study, we aimed to develop a viability qPCR for the selective detection of infectious and heat-treated RSIV using PMAxx dye and platinum compounds as survival markers. Furthermore, we optimized viability qPCR procedures for application in seawater through kinetic assays. The results of our study could help national quarantine agencies establish efficient disease control systems through the non-invasive detection of viruses.

## 2. Results

### 2.1. Initial Assessment of Viability Markers for qPCR Assays

**Figure 1:**
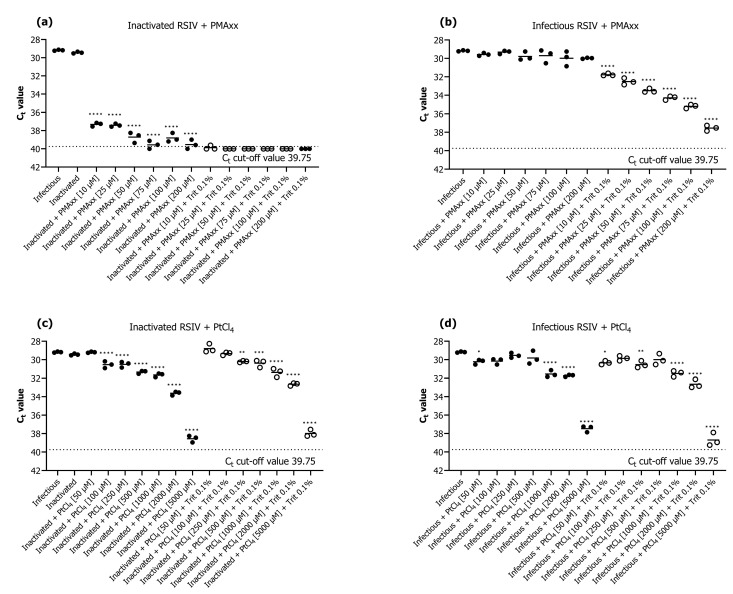
Evaluation of the performance of viability quantitative polymerase chain reaction (qPCR) for detection of inactivated and infectious red sea bream iridovirus (RSIV) using (**a**,**b**) propidium monoazide (PMAxx) dye and (**c**,**d**) platinum compounds (PtCl_4_). The black dotted line represents the cut-off cycle threshold (C_t_) values for qPCR. The solid line in each interval represents the average C_t_ value. Asterisks indicate significant differences from infectious RSIV without added viability markers for each viability marker concentration. * *p* < 0.05; ** *p* < 0.01; *** *p* < 0.001; **** *p* < 0.0001.

We investigated the efficiency of PMAxx and PtCl_4_ as the survival markers for qPCR assays. The results revealed that PMAxx prevented qPCR amplification of inactivated RSIV better than platinum compounds (Figure 1). The addition of only PMAxx at none of the tested concentrations (10–200 μM) inhibited the qPCR amplification of inactive or infective RSIV (Figure 1a,b). On the contrary, qPCR in both inactive or infective RSIV was inhibited in all groups when PMAxx (10–200 μM) was combined with 0.1% Triton X-100 (Figure 1a,b). Among the groups in which only PMAxx was added, the group treated with 75 μM PMAxx showed the highest qPCR inhibition—the C_t_ value significantly decreased by 10.38 compared to that of the infective RSIV C_t_ value (*p* < 0.0001; Figure 1a). This represents an approximately 1769-fold decrease from 10^5.1^ RSIV copies/200 μL to 10^1.8^ RSIV copies/200 μL. Therefore, the optimal concentration of PMAxx for RSIV survival qPCR was considered 75 μM and used in subsequent experiments. Furthermore, the addition of PtCl_4_ inhibited the qPCR of both inactive (at >100 μM) and infective (at >1000 μM) RSIV (Figure 1c,d).

### 2.2. Verification and Application of the PMAxx-Based Viability qPCR Assay

**Figure 2:**
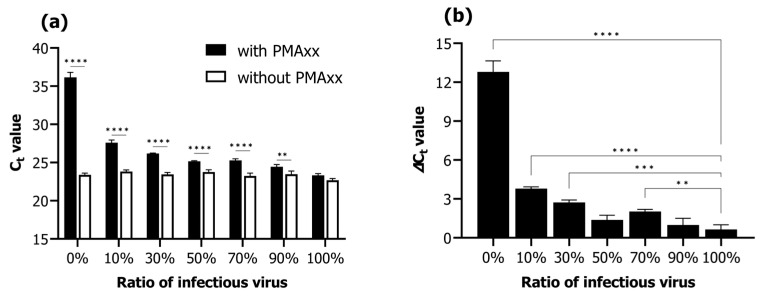
Viability qPCR results for selective detection of infectious RSIV in samples containing a mixture of infectious and inactive RSIV. qPCR was performed with or without the addition of PMAxx dye, depending on the proportion of infectious RSIV. (**a**) C_t_ values according to the RSIV infection rate and the presence or absence of PMAxx. (**b**) Delta C_t_ values according to the presence or absence of PMAxx. Delta C_t_ = C_t_ (with PMAxx) − C_t_ (without PMAxx). Asterisks indicate significant differences between the C_t_ values of viruses without added viability markers for each infectious RSIV ratio and the delta C_t_ values for different infectious RSIV ratios. ** *p* < 0.01; *** *p* < 0.001; **** *p* < 0.0001.

Viability qPCR was assessed according to the proportions of infectious and inactivated RSIV suspensions (infectious RSIV virus ratio; 0%, 10%, 30%, 50%, 70%, 90%, and 100%). Under conditions without the addition of PMAxx, the C_t_ values negligibly changed from 22.68 ± 0.24 C_t_ to 23.38 ± 0.24 C_t_ as the percentage of infectious virus decreased from 100% to 0% (Figure 2a). In contrast, under conditions where PMAxx was added, the C_t_ values increased notably from 23.32 ± 0.24 C_t_ to 36.17 ± 0.63 C_t_ as the percentage of infectious virus decreased from 100% to 0% (Figure 2a). Moreover, the C_t_ values in the presence of PMAxx were significantly different in all ratios of infectious RSIV (0–90%) except for that at 100% (*p* < 0.01; *p* < 0.0001; Figure 2a). In the 0% infectious RSIV ratio interval, the delta C_t_ value according to the presence or absence of PMAxx was 12.79 (Figure 2b). These results indicate that the PMAxx-based viability qPCR assay is suitable for selectively quantifying infectious RSIV.

### 2.3. Efficiency of Concentration of RSIV and Virus Isolation in Seawater

**Figure 3:**
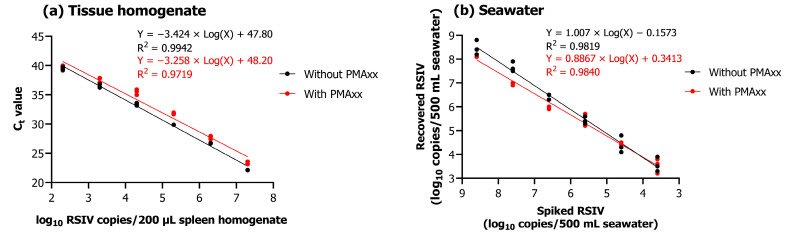
(**a**) qPCR and viability qPCR results obtained using 10-fold serially diluted RSIV-infected tissue homogenate. (**b**) Relationship between spiked RSIV and the number of copies recovered. Ten-fold serially diluted RSIV was concentrated based on iron flocculation, followed by qPCR and viability qPCR. All experiments were performed in triplicate. C_t_ values were plotted against RSIV copy numbers obtained using qPCR and viability qPCR; linear regression equations and correlation coefficients (R^2^) are shown in the graph.

Upon performing qPCR and PMAxx-based viability qPCR of 200 μL tissue homogenates of different concentrations (10^2.3^–10^7.3^ RSIV copies/200 µL), a linear relationship was observed between the C_t_ value and the concentration of 10-fold serially diluted RSIV (R^2^; without PMAxx, 0.9942; with PMAxx, 0.9719) (Figure 3a). Different concentrations of RSIV (10^1.6^–10^8.6^ RSIV copies/500 mL) were spiked into seawater and concentrated based on iron flocculation, followed by qPCR and PMAxx-based viability qPCR. As a result, a strong linear relationship was observed between spiked RSIV and the number of recovered RSIV copies (R^2^; without PMAxx, 0.9819; with PMAxx, 0.9840) (Figure 3b).

After performing iron-based flocculation, the quantitative detection limit of qPCR and viability qPCR was confirmed to be approximately 10^3.6^ RSIV copies/500 mL. Furthermore, the average recovery rate for the number of spikes within all concentration intervals was approximately 78.7% (Table 1). In the cell culture, RSIV-characteristic CPE was observed when seawater containing at least 10^4.6^ RSIV copies/500 mL was added (Table 1).

### 2.4. Analysis of RSIV Viability in Seawater Using the PMAxx-Based qPCR Assay

**Figure 4:**
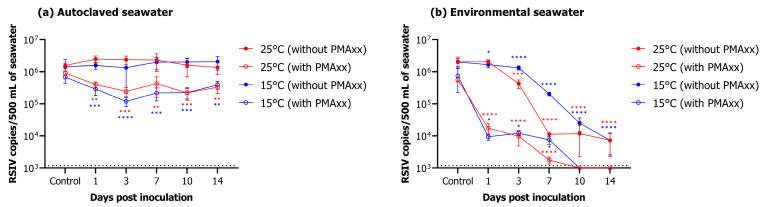
Changes in RSIV copy number under different marine environments. (**a**) Results of changes in RSIV viability in autoclaved seawater over time. (**b**) Results of changes in RSIV viability in environmental seawater over time. Error bars represent the standard deviation of the mean (*n* = 3). The dotted line represents the detection limit. Asterisks indicate significant differences (* *p* < 0.05, ** *p* < 0.01, *** *p* < 0.001, **** *p* < 0.0001) compared with the control.

In autoclaved seawater, RSIV was detected for up to 14 days after virus inoculation, regardless of water temperature (Figure 4a). In the group without PMAxx, RSIV copy number was 10^6.2^ copies/500 mL (control) at both 25 °C and 15 °C on day 0, and 10^6.1^ and 10^6.3^, respectively, on day 14, showing no difference (Figure 4a, Table 2). In the group to which PMAxx was added, RSIV copy number was 10^6.0^ and 10^5.8^ RSIV copies/500 mL at 25 °C and 15 °C, respectively, on day 0, and an insignificant decrease to 10^5.5^ and 10^5.6^ RSIV copies/500 mL, respectively, was observed on day 14 (Figure 4a, Table 2). In environmental seawater, the RSIV copy number decreased rapidly 3 days after RSIV inoculation in the group without PMAxx at 25 °C; the copy number was 10^3.9^ RSIV copies/500 mL on day 14 (Figure 4b, Table 2). For the same group, the decrease in copy number was slower at 15 °C than that at 25 °C. However, it gradually decreased after day 3, and on day 14, the RSIV copy number was 10^3.9^ copies/500 mL, similar to that at 25 °C. In the group inoculated with PMAxx, the number of RSIV copies decreased rapidly from 10^5.8^ RSIV copies/500 mL on day 0 (control) to 10^4.2^ copies/500 mL on day 1 at 25 °C in environmental seawater. Additionally, on day 7, the copy number was 10^3.2^ RSIV copies/500 mL. However, RSIV was not detected on day 10. Similarly, at 15 °C, the RSIV copy number decreased rapidly from day 1 after RSIV inoculation and was 10^3.9^ copies/500 mL on day 7. However, it was not detected from day 10 onwards (Figure 4b, Table 2).

The CPE in Pagrus major fin (PMF) cells was observed at all sampling times in RSIV cultured at 25 °C and 15 °C in the autoclaved seawater group. In environmental seawater, CPE was observed up to day 3 at 25 °C and up to day 7 at 15 °C (Table 2).

### 2.5. Viability Test of RSIV via Intraperitoneal Challenge Using Seawater

**Figure 5:**
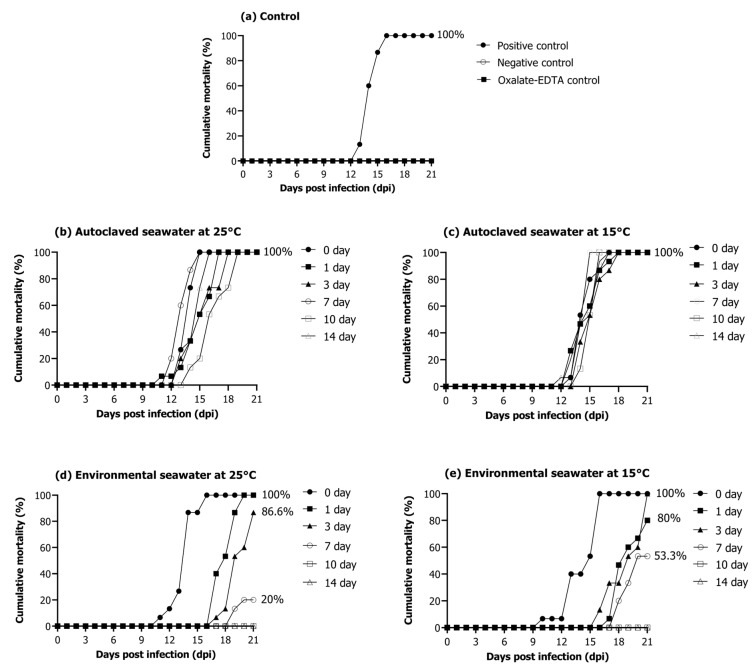
Cumulative mortality of rock bream after intraperitoneal injection of the resuspended virus after storing RSIV for different days in various seawater environments. (**a**) Positive control, 10^6.2^ RSIV copies/fish (cell culture); negative control, PBS; oxalate-EDTA control and oxalate-EDTA buffer 10-fold dilution were injected. (**b**,**c**) Injection of RSIV stored in autoclaved seawater at 25 °C and 15 °C. (**d**,**e**) Injection of RSIV stored in environmental seawater at 25 °C and 15 °C.

The positive control group injected with the RSIV cell culture showed 100% cumulative mortality for 3 weeks (Figure 5a). No mortality was observed in the negative control group injected with phosphate-buffered saline (PBS) and 10-fold diluted oxalate-EDTA (Figure 5a). In the autoclaved seawater (25 °C and 15 °C) groups, 100% mortality was observed in 3 weeks regardless of water temperature and storage period (Figure 5b,c). The group injected with RSIV stored in environmental seawater at 25 °C for 0 and 1 days showed 100% mortality within 3 weeks after injection. Furthermore, this group showed a cumulative mortality of 86.6% on day 3 and 20% on day 7 (Figure 5d). Mortality was not observed in groups injected with RSIV stored in environmental seawater for 10 and 14 days. The groups injected with RSIV stored in environmental seawater at 15 °C for 0 and 1 days showed a cumulative mortality of 100% and 80%, respectively, after injection of the RSIV suspension (Figure 5e). The groups injected with RSIV stored in environmental seawater for 3 and 7 days showed 100% and 53.3% cumulative mortality, respectively, within 3 weeks of injection. There was no mortality in the groups injected with RSIV stored in environmental seawater for 10 and 14 days.

## 3. Discussion

Horizontal transmission through water is one of the most important transmission routes for aquatic viruses [3,7,8]. However, viruses in the natural environment are inactivated by several factors, including physicochemical parameters or the bioload of water [24]. This study aimed to provide an efficient disease control system that selectively detects and quantifies potentially infectious RSIV in a shorter time than that required by conventional cell culture-based assays. We evaluated PMAxx and platinum compounds as viability markers that can be applied prior to nucleic acid extraction to prevent DNA amplification from an inactivated virus, allowing only the infectious virus to be amplified in the qPCR assay. We selected PMAxx as the best-performing survival marker. Additionally, the viability qPCR using PMAxx efficiently discriminated and detected infectious RSIV. We further demonstrated that this assay could better analyze the viability of RSIV in seawater than the conventional qPCR and cell culture methods.

Propidium monoazide concentration is a key factor influencing the efficiency of viability qPCR to differentiate between infectious and inactive viruses [25]. In this study, the optimal concentration of PMAxx that effectively inhibited the amplification of inactive viruses without affecting infectious viruses was 75 μM. Several previous studies have reported that adding nonionic surfactants, such as Triton X-100 and sodium dodecyl sulfate, during the pretreatment of PMAxx qPCR can increase the permeability of the PMA dye to partially or completely disrupt viruses [26,27,28]. We attempted to add Triton X-100 to improve the penetration efficiency of PMAxx infectious or inactivated RSIV. However, PMAxx-based qPCR was inhibited when infectious RSIV was pretreated with Triton X-100. Previous studies have shown that surfactants dissolve the lipid bilayer of enveloped viruses (e.g., influenza viruses) [29]. Therefore, surfactants should not be used with viability markers to assess the infectivity of RSIV, an enveloped virus [29]. Non-enveloped viruses may not be affected by surfactants because they do not contain a lipid bilayer membrane in their structure. Based on these results, 75 μM PMAxx without Triton X-100 as a surfactant was selected as the most effective method and used for further analysis in this study.

In our study, PtCl_4_, as a survival marker for RSIV, showed negative results. A previous study reported that PtCl_4_ at 2500 μM inhibited the qPCR of the inactivated virus without affecting that of the infectious murine norovirus (MNV) [30], wherein at a lower concentration (500 μM), it was suitable for testing the viability of other viruses, such as porcine epidemic diarrhea virus or hepatitis E virus [19,31]. In contrast, Chen et al. [32] reported a higher potential of PMAxx than PtCl_4_ in distinguishing inactivated noroviruses. The inconsistency in these results could be attributed to the differences in experimental conditions, such as inactivation methods and target viruses tested in these studies. It has been reported that PtCl_4_ penetrates living cells in small amounts and causes a slight decrease in the qPCR fluorescence signal. Furthermore, unlike PMAxx, it does not react with water molecules and becomes inactivated after reacting with viruses [33]. The suppression of qPCR after PtCl_4_ treatment in this study could be because the PtCl_4_ molecules were not eliminated and modified the DNA during the extraction procedure [33]. Our study of RSIV viability via qPCR using platinum compounds is valuable, considering that no such studies have been conducted previously.

The reliability of the PMAxx-based viability qPCR was confirmed. When RSIV was detected without adding PMAxx, no significant difference in C_t_ values occurred among the suspensions with different percentages of infectious virus (0–100%). However, C_t_ and delta C_t_ values increased significantly as the proportion of infectious RSIV decreased after PMAxx pretreatment. Although viability qPCR did not completely suppress the signal of 100% heat-treated RSIV, it suppressed the signal approximately 16,670-fold from 10^7.3^ RSIV copies/200 μL (infectious RSIV) to 10^2.9^ RSIV copies/200 μL (inactivated RSIV). The RSIV used in this study had a fairly low C_t_ value, i.e., a high copy number. Therefore, it is possible that not all samples were heat inactivated or that the inactivated virus particles aggregated and prevented PMAxx penetration through the damaged virus [34]. This observation is consistent with the findings of previous studies showing that the effectiveness of viability markers is increased in samples containing low virus concentrations [35,36,37].

The viability qPCR of RSIV in seawater was successfully confirmed. In our study, the oxalic acid-EDTA buffer was used for concentrating seawater. However, this stock solution is highly toxic to cells and fish. The virus suspension was diluted 10-fold with PBS to inoculate the concentrated virus into cells. However, since the virus copy number decreased with dilution, a 10-fold higher RSIV suspension was prepared and introduced into seawater. In experiments evaluating RSIV infectivity in seawater, CPE in cells showed a 10- or 100-fold lower detection limit than that in the viability qPCR. The viability qPCR method reduces the risk of underestimating RSIV present in seawater because samples concentrated in seawater are used directly without further dilution. In addition, viability qPCR is a better method than infectivity assessment through cell inoculation because it is fast and requires no special skills.

In this study, we validated the optimized viability qPCR for selective detection of RSIV infectivity in seawater by performing qPCR in various seawater conditions. Furthermore, the cell inoculation and rock bream artificial infection experiments were performed to validate the reliability of the viability qPCR results. Water temperature is often recognized as the most influential factor in virus infectivity; a higher water temperature leads to faster virus inactivation [38,39]. In this study, RSIV stored in autoclaved seawater maintained the infectivity for up to 14 days regardless of water temperature. On the contrary, the infectivity of RSIV stored in environmental seawater decreased more rapidly at 25 °C than at 15 °C. Consistent with our study, a previous study demonstrated that nervous necrosis virus infectivity in seawater was maintained for a longer period at 15 °C than at 25 °C, demonstrating an inverse correlation between viral infectivity and water temperature elevation [40]. Furthermore, our results demonstrated that RSIV stored in environmental seawater lost infectivity faster than that stored in microbial-free autoclaved seawater. This could be attributed to the presence of microflora, especially bacterial populations with an antiviral activity that affect virus survival in seawater [41,42,43]. Although we did not investigate the bacterial populations in the environmental seawater used in our study, the presence of microbial communities and their contribution to virus survival cannot be ruled out. Therefore, further studies are needed to clarify the role of microbial communities, such as bacteria, in RSIV viability.

In environmental seawater, qPCR analysis without the addition of PMAxx confirmed that RSIV was detected up to 14 days at 25 °C and 15 °C. However, viability qPCR and rock bream artificial infection experiments revealed that RSIV stored in environmental seawater at 25 °C and 15 °C showed no virus detection or fish mortality after 7 days. Moreover, no CPE was observed after 3–7 days in the cell inoculation experiments. This observation indicates that RSIV can be inactivated within approximately 7–10 days in seawater, demonstrating that positive results via conventional qPCR in seawater overestimate the disease risk.

The three viability experiments we analyzed can be used to assess RSIV infectivity. However, the cell inoculation experiment is limited by its long-time requirement, absence of cell lines, low susceptibility to viruses, and experimental techniques. Rock bream is known to be the most sensitive fish species to RSIV [3,44], but the confirmation of virus infectivity through fish has various limitations, such as laboratory facilities and biological consumptions. Therefore, we inferred that the viability qPCR assay we developed in this study is one of the suitable alternative methods to confirm viral infectivity at a low cost and in a short time.

Our study results demonstrate the potential of viability qPCR as a non-invasive method to assess the risk for RSIV occurrence in actual farm seawater. The findings suggest that the viability qPCR to detect viruses in seawater can help the epidemiological analysis of disease outbreaks. Furthermore, it can help estimate the risk of transmission between farms through disease spread model simulation or by building a disease spread forecasting system for farms around farms with RSIVD outbreaks [45]. Previous studies detected approximately 10^3^–10^6^ RSIV copies/L seawater from red sea bream farms. Furthermore, in winter, RSIV was detected in seawater at a low water temperature of 11.7 °C [9]. However, studies on the infectivity of the detected virus are lacking. The detection limit of our viability qPCR method was similar to the RSIV load present in the seawater of the farm, demonstrating its applicability in the aquaculture field. To selectively detect only infectious RSIV in fish farm seawater, further studies on the application and reproducibility of viability qPCR should be performed.

## 4. Materials and Methods

### 4.1. Virus Preparation

Red sea bream iridovirus was obtained from RSIV-infected rock bream spleen tissue and replicated in the PMF cell line (National Fishery Products Quality Management Service, Busan, Republic of Korea) [6,46]. The PMF cell line was cultured at 25 °C in L-15 medium (Gibco, Billings, MT, USA) supplemented with 10% fetal bovine serum (FBS; Gibco) and 1% antibiotic-antimycotic (A-A; 100 U/mL penicillin, 100 μg/mL streptomycin, and 25 μg/mL amphotericin B, Gibco). The RSIV-infected rock bream spleen tissue was homogenized in L-15 medium and centrifuged at 10,000× *g* for 5 min. Then, the supernatant was collected and filtered through a 0.22 μm syringe filter. Subsequently, 1 mL of the supernatant was inoculated into the PMF cell line. After 4 h of infection, the culture medium was replaced with fresh L-15 medium and cultured at 25 °C for 7 days. Finally, the harvested supernatant was used as an inoculum in subsequent experiments.

### 4.2. Viral DNA Extraction and RSIV Detection

Viral DNA was extracted using the AccuPrep^®^ Genomic DNA Extraction Kit (Bioneer, Daejeon, Republic of Korea) according to the manufacturer’s instructions. The quantitative analysis of RSIV was performed using a previously reported qPCR assay [14]. The final volume of the reaction mixture was 25 μL comprising 12.5 μL HS Prime qPCR Premix with UDG (2×) (Genetbio, Daejeon, Republic of Korea), 900 nM of each primer, a 250 nM probe, and 5 μL DNA. The thermal cycling conditions consisted of 45 cycles of 5 s at 95 °C followed by 10 s at 60 °C, with one cycle lasting 1 min at 95 °C. A cut-off value of 39.75 cycle threshold (C_t_) was employed for quantitative analysis using the same real-time PCR equipment and analysis reagents as described in a previous study [14].

### 4.3. Optimization of Viability Marker Concentrations

Photoactivatable dyes, such as PMAxx (Biotium, Hayward, CA, USA), and platinum compounds, such as platinum (IV) chloride (PtCl_4_; Sigma-Aldrich, St. Louis, MO, USA), were used as viability markers in this study. Propidium monoazide was diluted to a 5 mM concentration in nuclease-free water. Afterward, PtCl_4_ was diluted to 500 mM using dimethylsulfoxide and further diluted to 50 mM in nuclease-free water. All viability marker stock solutions were stored at −20 °C in the dark. Infectious RSIV (approximately 10^4^ RSIV copies/200 μL) was obtained using the virus suspension obtained via the method described in Section 4.1. Subsequently, 200 μL of the virus suspension was dispensed into an Eppendorf tube for virus inactivation and then heated in a 95 °C heat block for 30 min.

For the viability assays, the photoactive PMAxx dye was added to the infectious or inactivated virus suspension at final concentrations of 10, 25, 50, 75, 100, and 200 μM. Photoactivation of the PMAxx dye was performed by incubation in the dark at 20 °C in an orbital shaker (150 rpm) for 30 min, followed by exposure to a light emitting diode (LED) light for 30 min in a photoactivation system (PMA-Lite™ LED Photolysis Device, Biotium). PtCl_4_ was added at final concentrations of 50, 100, 250, 500, 1000, 2000, and 5000 μM to the infectious or inactive virus suspensions, followed by incubation for 30 min at 20 °C in an orbital shaker (150 rpm). Additionally, 0.1% Triton X-100 (Sigma-Aldrich, USA), which is known to increase virus permeability under all survival marker conditions, was added, and viability assays were performed [26,27,28]. Each experiment comprised a viral suspension sample without viability markers as a positive control, and all experiments were performed in triplicate. After viability marker treatment, RSIV DNA was extracted, as described above, for amplification using viability qPCR.

### 4.4. Selective Detection of Infectious RSIV

Propidium monoazide was selected as the most reliable viability marker and was used in this study to evaluate the performance of survival qPCR. To evaluate the performance of the PMAxx-based viability qPCR assay to selectively detect infectious RSIV in the presence of non-infectious RSIV, the proportion of infectious RSIV in a 200 μL virus suspension was set as 0%, 10%, 30%, 50%, 70%, 90%, or 100% (the remaining proportion was that of the heat-treated RSIV), and then PMAxx-based viability qPCR was performed. Different samples with the above-mentioned concentrations were prepared. Furthermore, qPCR was performed without adding PMAxx. All qPCR experiments were performed in triplicate for all samples. Then, the delta C_t_ values of PMAxx-treated and PMAxx-untreated samples were analyzed according to the infectious virus ratio.

### 4.5. Concentration of RSIV in Seawater

A previously reported iron-based flocculation method was used to concentrate the virus in seawater, with slight modifications [47]. Approximately 4.83 g of iron(III) chloride hexahydrate (FeCl_3_∙6H_2_O) was dissolved in 100 mL distilled water to form a Fe-virus flocculate using iron chloride. Subsequently, 50 µL of the iron chloride solution was added to 500 mL seawater. The seawater was gently stirred (200 rpm) for 1 h at 20 °C using a magnetic stirrer. The resulting Fe-virus flocculates were filtered under reduced pressure through a 0.8 μm pore size polycarbonate filter (Whatman, Maidstone, UK) held to a filter holder with a receiver (Nalgene, New York, NY, USA). The filter in which the Fe-virus flocculates were captured was transferred to a 5 mL round-bottom tube, and 1 mL oxalate-EDTA buffer was added, as previously reported [48]. Virus resuspension was performed in the dark at 4 °C for 4 h using an MX-RD/RL-Pro LCD Digital Rotator (40 rpm; Dragonlab, Beijing, China).

### 4.6. Validation of the Method for RSIV Detection in Seawater

Red sea bream iridovirus-infected rock bream spleens were homogenized in PBS and centrifuged at 10,000× *g* and 4 °C for 10 min. After centrifugation, the supernatant was filtered through a 0.45 μm syringe filter and sequentially diluted 10-fold to prepare viruses of various concentrations, ranging from 10^2.3^ to 10^7.3^ RSIV copies/200 μL. Afterward, PMAxx-based viability qPCR and qPCR were performed, as mentioned above.

Serially diluted RSIV suspensions were spiked into seawater (500 mL) at various concentrations, ranging from 10^1.6^ to 10^8.6^ RSIV copies/500 mL. Subsequently, the seawater sample to which RSIV was added was first filtered through a glass microfiber filter with a 1.6 μm pore size (GF/A; Whatman) and then through a 0.45 μm pore MF-Millipore™ membrane filter (Merck, Darmstadt, Germany). Afterward, 50 μL of the previously prepared iron chloride solution was added to the filtered RSIV spiked seawater and gently stirred (200 rpm) for 1 h at 20 °C using a magnetic stirrer. Virus flocculation and resuspension were performed as described above. Three replicates were prepared for each concentration. Next, virus quantification was performed after DNA extraction, as described above, using a resuspended solution of 1 mL concentrated RSIV.

Owing to the cytotoxicity of the buffer, the virus resuspended in oxalate-EDTA buffer was diluted 10-fold in PBS to determine the intracellular CPE of the concentrated virus. Thus, a concentration of 10 times the viral seawater concentration described above was inoculated. One milliliter of the viral dilution was inoculated onto a PMF cell monolayer cultured in L-15 medium supplemented with 1% FBS and 1% A-A. After 4 h, the inoculum was removed, washed once with Dulbecco’s PBS, and replaced with fresh medium. Cytopathic effects were observed for 7 days.

### 4.7. Virus Viability in Seawater

#### 4.7.1. Quantitative Polymerase Chain Reaction and Viability qPCR Analysis

Seawater was collected from the coast of Tongyeong, Gyeongsangnam-do, Republic of Korea. Before analysis, seawater was filtered using a 0.22 μm pore size syringe filter and sterilized in an autoclave. Environmental seawater was used for follow-up research immediately after transporting the collected seawater to the laboratory. Red sea bream iridovirus was inoculated into 1 L capped and clear glass bottles containing 500 mL seawater (autoclave or environmental seawater) and incubated at 25 °C and 15 °C, respectively. Virus concentration at the start of all analyses was 10^6.2^ RSIV copies/mL. After virus inoculation, samples were taken on days 0 (control), 1, 3, 7, 10, and 14. After concentrating the seawater, qPCR and viability qPCR were performed as described in Section 4.2.

#### 4.7.2. Cell Inoculation

The viability of RSIV in seawater was confirmed by CPE after inoculation of the virus concentrates into PMF cells. A virus suspension with a concentration (10^7.2^ RSIV copies/mL) 10 times higher than the RSIV inoculation concentration mentioned above was inoculated into autoclaved seawater and environmental seawater. After incubation at 25 °C and 15 °C, respectively, the virus was concentrated for 0, 1, 3, 7, 10, and 14 days. Subsequently, it was diluted 10-fold with PBS. The environmental seawater was filtered through a 0.45 μm pore size syringe filter and inoculated into a PMF cell monolayer.

#### 4.7.3. Fish Challenge Experiment

To compare RSIV viability in various seawater conditions, rock breams were inoculated with the resuspended virus. All experimental fish were purchased from a rock bream hatchery located in Geoje, Gyeongsangnam-do, Republic of Korea, where RSIVD has not been reported. Approximately 800 rock breams (length: 10.9 ± 3.6 cm, weight: 22.4 ± 7.2 g) were acclimatized in a 1600 L tank for 2 weeks. The tank was a flow-through aquaculture system. Furthermore, it was continuously supplied with sand-filtered, 50 μm filter-housed, and ultraviolet (UV)-treated (>30 mW/cm^2^) seawater (500–1000 L/h). For virus inoculation, RSIV was inoculated by 10^8.2^ RSIV copies/mL in 500 mL of environmental and autoclaved seawater, respectively, according to the concentrations obtained through iron flocculation described in Section 4.5, and the resuspended virus was diluted 10-fold in PBS.

Rock breams were housed in each group (*n* = 15) after maintaining a 200 L tank at 25 °C. In the RSIV viability infection experiment, 0.1 mL (10^6.2^ RSIV copies/fish) of the resuspended RSIV 10-fold dilution was intraperitoneally injected into each rock bream. In the positive control group, 10^6.2^ RSIV copies/fish (cell culture) was intraperitoneally injected. Furthermore, the oxalate-EDTA control group (virus-free group) was intraperitoneally injected with 0.1 mL oxalate-EDTA buffer diluted 10-fold with PBS. The negative control group was intraperitoneally injected with the same amount of PBS. Rock breams were fed a commercial feed once a day, and they were continuously supplied with sand-filtered, 1 μm filter-housed, and UV-treated (>30 mW/cm^2^) seawater (100 L/h). Cumulative mortality was observed for 3 weeks.

All experimental protocols followed the ethical guidelines of the Institutional Animal Care and Use Committee of the Gyeongsang National University and were approved by the same committee (approval number: GNU-220526-E0056).

### 4.8. Statistical Analysis

Statistical tests were performed using GraphPad Prism 9.5 (https://www.graphpad.com/scientific-software/prism/; accessed on 7 December 2022). Significant differences according to survival marker concentrations were evaluated using one-way analysis of variance (ANOVA) and Dunnett’s correction based on infectious RSIV. When the C_t_ values were compared according to infectious RSIV rates, statistical analysis was performed using two-way ANOVA. Furthermore, significant differences in the delta C_t_ values among groups were analyzed using one-way ANOVA. Significant differences in RSIV viability over time compared to the control in seawater were assessed using Dunnett’s correction based on one-way ANOVA. A *p*-value of <0.05 was considered statistically significant.

## Figures and Tables

**Table 1:** Recovery of red sea bream iridovirus (RSIV) from experimentally virus-spiked seawater, as determined using quantitative polymerase chain reaction (qPCR), viability qPCR, and cell culture isolation.

Spiked Virus (RSIV Copies/500 mL)	qPCR Results (without PMAxx)				Viability qPCR Results (with PMAxx)				CPE ^a^
	Recovered Virus (RSIV Copies/500 mL)				Recovered Virus (RSIV Copies/500 mL)				
	1	2	3	Mean	1	2	3	Mean	
10^8.6^	10^8.8^	10^8.4^	10^8.2^	10^8.5^	10^8.2^	10^8.1^	10^8.1^	10^8.1^	+
10^7.6^	10^7.6^	10^7.5^	10^7.9^	10^7.7^	10^7.0^	10^6.9^	10^7.0^	10^7.0^	+
10^6.6^	10^6.3^	10^6.5^	10^6.3^	10^6.4^	10^5.9^	10^6.0^	10^6.0^	10^6.0^	+
10^5.6^	10^5.3^	10^5.6^	10^5.4^	10^5.5^	10^5.4^	10^5.2^	10^5.7^	10^5.5^	+
10^4.6^	10^4.8^	10^4.3^	10^4.1^	10^4.5^	10^4.4^	10^4.5^	10^4.5^	10^4.5^	+/−
10^3.6^	10^3.5^	10^3.9^	10^3.3^	10^3.6^	10^3.6^	10^3.2^	10^3.8^	10^3.6^	−
10^2.6^	ND ^b^	ND	ND	ND	ND	ND	ND	ND	−
10^1.6^	ND	ND	ND	ND	ND	ND	ND	ND	−

^a^ +, complete cytopathic effect (CPE); +/−, partial CPE; −, no CPE was observed. ^b^ not detected or undetermined based on the 95% limit of detection (LoD_95%_) for qPCR. PMAxx, propidium monoazide.

**Table 2:** Results of RSIV qPCR, viability qPCR, and cell culture isolation under various seawater environments. Results represent the average of triplicates per assay.

Days	Autoclaved Seawater						Environmental Seawater					
	25 °C			15 °C			25 °C			15 °C		
	Without PMAxx	With PMAxx	CPE ^a^	Without PMAxx	With PMAxx	CPE	Without PMAxx	With PMAxx	CPE	Without PMAxx	With PMAxx	CPE
0	10^6.2 b^	10^6.0^	+	10^6.2^	10^5.8^	+	10^6.3^	10^5.8^	+	10^6.3^	10^5.9^	+
1	10^6.4^	10^5.6^ **	+	10^6.2^	10^5.5^ ***	+	10^6.3^	10^4.2^ ****	+	10^6.2^ *	10^4.0^ *	+
3	10^6.4^	10^5.4^ ***	+	10^6.1^	10^5.1^ ****	+	10^5.6^ ***	10^4.0^ ****	+/−	10^6.1^ ****	10^4.1^ *	+/−
7	10^6.4^	10^5.6^ **	+	10^6.3^	10^5.3^ ***	+	10^4.1^ ****	10^3.2^ ****	−	10^5.3^ ****	10^3.9^ *	+/−
10	10^6.2^	10^5.3^ ***	+	10^6.3^	10^5.3^ ***	+	10^4.1^ ****	ND ^c^	−	10^4.4^ ****	ND	−
14	10^6.1^	10^5.5^ **	+	10^6.3^	10^5.6^ **	+	10^3.9^ ****	ND	−	10^3.9^ ****	ND	−

^a^ +, complete CPE; +/−, partial CPE; −, no CPE was observed. ^b^ RSIV copies/500 mL. ^c^ not detected or undetermined based on the LoD^95%^ for qPCR. Asterisks indicate significant differences (* *p* < 0.05, ** *p* < 0.01, *** *p* < 0.001, **** *p* < 0.0001) compared with the control.

## Data Availability

Not applicable.

## References

[1] Kurita J., Nakajima K. (2012). Megalocytiviruses. Viruses.

[2] Inouye K., Yamano K., Maeno Y., Nakajima K., Matsuoka M., Wada Y., Sorimachi M. (1992). Iridovirus infection of cultured red sea bream, *Pagrus major*. Fish Pathol..

[3] WOAH (World Organisation for Animal Health) (2012). Red sea bream iridoviral disease. Manual of Diagnostic Tests for Aquatic Animals.

[4] Sohn S.G., Choi D.L., Do J.W., Hwang J.Y., Park J.W. (2000). Mass mortalities of cultured striped beakperch, *Oplegnathus fasciatus* by iridoviral infection. J. Fish Pathol..

[5] Kim Y.J., Jung S.J., Choi T.J., Kim H.R., Rajendran K.V., Oh M.J. (2002). PCR amplification and sequence analysis of irido-like virus infecting fish in Korea. J. Fish Dis..

[6] Kim K.H., Choi K.M., Joo M.S., Kang G., Woo W.S., Sohn M.Y., Son H.J., Kwon M.G., Kim J.O., Kim D.H. (2022). Red sea bream iridovirus (RSIV) kinetics in rock bream (*Oplegnathus fasciatus*) at various fish-rearing seawater temperatures. Animals.

[7] He J.G., Zeng K., Weng S.P., Chan S.-M. (2002). Experimental transmission, pathogenicity and physical-chemical properties of infectious spleen and kidney necrosis virus (ISKNV). Aquaculture.

[8] Min J.G., Jeong Y.J., Jeong M.A., Kim J.O., Hwang J.Y., Kwon M.G., Kim K.I. (2021). Experimental transmission of red sea bream iridovirus (RSIV) between rock bream (*Oplegnathus fasciatus*) and rockfish (*Sebastes schlegelii*). J. Fish Pathol..

[9] Kawato Y., Mekata T., Inada M., Ito T. (2021). Application of environmental DNA for monitoring red sea bream iridovirus at a fish farm. Microbiol. Spectr..

[10] Clem L.W., Moewus L., Sigel M.M. (1961). Studies with cells from marine fish in tissue culture. Proc. Soc. Exp. Biol. Med..

[11] Caipang C.M., Haraguchi I., Ohira T., Hirono I., Aoki T. (2004). Rapid detection of a fish iridovirus using loop-mediated isothermal amplification (LAMP). J. Virol. Methods.

[12] Kurita J., Nakajima K., Hirono I., Aoki T. (1998). Polymerase chain reaction (PCR) amplification of DNA of red sea bream iridovirus (RSIV). Fish Pathol..

[13] Choi S.K., Kwon S.R., Nam Y.K., Kim S.K., Kim K.H. (2006). Organ distribution of red sea bream iridovirus (RSIV) DNA in asymptomatic yearling and fingerling rock bream (*Oplegnathus fasciatus*) and effects of water temperature on transition of RSIV into acute phase. Aquaculture.

[14] Kim K.H., Choi K.M., Kang G., Woo W.S., Sohn M.Y., Son H.J., Yun D., Kim D.H., Park C.I. (2022). Development and validation of a quantitative polymerase chain reaction assay for the detection of red sea bream iridovirus. Fishes.

[15] Ito T., Yoshiura Y., Kamaishi T., Yoshida K., Nakajima K. (2013). Prevalence of red sea bream iridovirus among organs of Japanese amberjack (*Seriola quinqueradiata*) exposed to cultured red sea bream iridovirus. J. Gen. Virol..

[16] Shirasaki N., Matsushita T., Matsui Y., Koriki S. (2020). Suitability of pepper mild mottle virus as a human enteric virus surrogate for assessing the efficacy of thermal or free-chlorine disinfection processes by using infectivity assays and enhanced viability PCR. Water Res..

[17] Razafimahefa R.M., Ludwig-Begall L.F., Le Guyader F.S., Farnir F., Mauroy A., Thiry E. (2021). Optimisation of a PMAxx™-RT-qPCR assay and the preceding extraction method to selectively detect infectious murine norovirus particles in mussels. Food Environ. Virol..

[18] Cuevas-Ferrando E., Randazzo W., Pérez-Cataluña A., Falcó I., Navarro D., Martin-Latil S., Díaz-Reolid A., Girón-Guzmán I., Allende A., Sánchez G. (2021). Platinum chloride-based viability RT-qPCR for SARS-CoV-2 detection in complex samples. Sci. Rep..

[19] Randazzo W., Vasquez-García A., Aznar R., Sánchez G. (2018). Viability RT-qPCR to distinguish between HEV and HAV with intact and altered capsids. Front. Microbiol..

[20] Canh V.D., Torii S., Yasui M., Kyuwa S., Katayama H. (2021). Capsid integrity RT-qPCR for the selective detection of intact SARS-CoV-2 in wastewater. Sci. Total Environ..

[21] Elizaquível P., Aznar R., Sánchez G. (2014). Recent developments in the use of viability dyes and quantitative PCR in the food microbiology field. J. Appl. Microbiol..

[22] Golpayegani A., Douraghi M., Rezaei F., Alimohammadi M., Nodehi R.N. (2019). Propidium monoazide-quantitative polymerase chain reaction (PMA-qPCR) assay for rapid detection of viable and viable but non-culturable (VBNC) *Pseudomonas aeruginosa* in swimming pools. J. Environ. Health Sci. Eng..

[23] Lee H.W., Lee H.M., Yoon S.R., Kim S.H., Ha J.H. (2018). Pretreatment with propidium monoazide/sodium lauroyl sarcosinate improves discrimination of infectious waterborne virus by RT-qPCR combined with magnetic separation. Environ. Pollut..

[24] Oidtmann B., Dixon P., Way K., Joiner C., Bayley A.E. (2017). Risk of waterborne virus spread—Review of survival of relevant fish and crustacean viruses in the aquatic environment and implications for control measures. Rev. Aquac..

[25] Zhang J., Khan S., Chousalkar K.K. (2020). Development of PMAxxTM-based qPCR for the quantification of viable and non-viable load of *Salmonella* from poultry environment. Front. Microbiol..

[26] Coudray-Meunier C., Fraisse A., Martin-Latil S., Guillier L., Perelle S. (2013). Discrimination of infectious hepatitis a virus and rotavirus by combining dyes and surfactants with RT-qPCR. BMC Microbiol..

[27] Dong L., Liu H., Meng L., Xing M., Wang J., Wang C., Chen H., Zheng N. (2018). Quantitative PCR coupled with sodium dodecyl sulfate and propidium monoazide for detection of viable *Staphylococcus aureus* in milk. J. Dairy Sci..

[28] Zhao Y., Chen H., Liu H., Cai J., Meng L., Dong L., Zheng N., Wang J., Wang C. (2019). Quantitative polymerase chain reaction coupled with sodium dodecyl sulfate and propidium monoazide for detection of viable *Streptococcus agalactiae* in milk. Front. Microbiol..

[29] Kawahara T., Akiba I., Sakou M., Sakaguchi T., Taniguchi H. (2018). Inactivation of human and avian influenza viruses by potassium oleate of natural soap component through exothermic interaction. PLoS ONE.

[30] Fraisse A., Niveau F., Hennechart-Collette C., Coudray-Meunier C., Martin-Latil S., Perelle S. (2018). Discrimination of infectious and heat-treated norovirus by combining platinum compounds and real-time RT-PCR. Int. J. Food Microbiol..

[31] Puente H., Randazzo W., Falcó I., Carvajal A., Sánchez G. (2020). Rapid Selective Detection of Potentially Infectious Porcine Epidemic Diarrhea Coronavirus Exposed to Heat Treatments Using Viability RT-qPCR. Front. Microbiol..

[32] Chen J., Wu X., Sanchez G., Randazzo W. (2020). Viability RT-qPCR to detect potentially infectious enteric viruses on heat-processed berries. Food Control.

[33] Cechova M., Beinhauerova M., Babak V., Slana I., Kralik P. (2021). A novel approach to the viability determination of *Mycobacterium avium* subsp. paratuberculosis using platinum compounds in combination with quantitative PCR. Front. Microbiol..

[34] Baert L., Wobus C.E., Van Coillie E., Thackray L.B., Debevere J., Uyttendaele M. (2008). Detection of murine norovirus 1 by using plaque assay, transfection assay, and real-time reverse transcription-PCR before and after heat exposure. Appl. Environ. Microbiol..

[35] Parshionikar S., Laseke I., Fout G.S. (2010). Use of propidium monoazide in reverse transcriptase PCR to distinguish between infectious and noninfectious enteric viruses in water samples. Appl. Environ. Microbiol..

[36] Kim S.Y., Ko G. (2012). Using propidium monoazide to distinguish between viable and nonviable bacteria, MS2 and murine norovirus. Lett. Appl. Microbiol..

[37] Gyawali P., Hewitt J. (2018). Detection of infectious noroviruses from wastewater and seawater using PEMAX^TM^ treatment combined with RT-qPCR. Water.

[38] Yates M.V., Gerba C.P., Kelley L.M. (1985). Virus persistence in groundwater. Appl. Environ. Microbiol..

[39] Lo S., Gilbert J., Hetrick F. (1976). Stability of human enteroviruses in estuarine and marine waters. Appl. Environ. Microbiol..

[40] Vázquez-Salgado L., Olveira J.G., Bandín I. (2022). Nervous necrosis virus viability modulation by water salinity and temperature. J. Fish Dis..

[41] Kamei Y., Yoshimizu M., Ezura Y., Kimura T. (1988). Screening of bacteria with antiviral activity from fresh water salmonid hatcheries. Microbiol. Immunol..

[42] Shimizu T., Yoshida N., Kasai H., Yoshimizu M. (2006). Survival of koi herpesvirus (KHV) in environmental water. Fish Pathol..

[43] Teng Y.F., Xu L., Wei M.Y., Wang C.Y., Gu Y.C., Shao C.L. (2020). Recent progresses in marine microbial-derived antiviral natural products. Arch. Pharm. Res..

[44] Kwon W.J., Choi J.C., Hong S., Kim Y.C., Jeong M.G., Min J.G., Jeong J.B., Kim K.I., Jeong H.D. (2020). Development of a high-dose vaccine formulation for prevention of megalocytivirus infection in rock bream (*Oplegnathus fasciatus*). Vaccine.

[45] Foreman M.G., Guo M., Garver K.A., Stucchi D., Chandler P., Wan D., Morrison J., Tuele D. (2015). Modelling infectious hematopoietic necrosis virus dispersion from marine salmon farms in the Discovery Islands, British Columbia, Canada. PLoS ONE.

[46] Kwon W.J., Yoon M.J., Jin J.W., Kim K.I., Kim Y.C., Hong S., Jeong H.D. (2020). Development and characterization of megalocytivirus persistently-infected cell cultures for high yield of virus. Tissue and Cell.

[47] John S.G., Mendez C.B., Deng L., Poulos B., Kauffman A.K.M., Kern S., Brum J., Polz M.F., Boyle E.A., Sullivan M.B. (2010). A simple and efficient method for concentration of ocean viruses by chemical flocculation. Environ. Microbiol. Rep..

[48] Kim M.J., Baek E.J., Kim K.I. (2022). Application of iron flocculation to concentrate white spot syndrome virus in seawater. J. Virol. Methods.

